# Co-registration of 3D echo and MR data to create physical models of congenital heart malformations

**DOI:** 10.1186/1532-429X-17-S1-P198

**Published:** 2015-02-03

**Authors:** Tyler Moore, Erin J Madriago, Eric S Renteria, Cristina Fuss, David J Sahn, Del Shepard, Michael Silberbach

**Affiliations:** Division, Pediatric Cardiology, Oregon Health & Sciences University, Portland, OR USA; Innovation Suite, Applications Engineer, Materialise, Plymouth, MI USA; Department of Radiology, Oregon Health & Sciences University, Portland, OR USA

## Background

MR reconstruction of virtual 3 dimensional volumes are routinely used to demonstrate cardiac malformations, particularly the complex geometric relationships between the great arteries and cardiac chambers. However, because of the need to signal average over multiple heart beats, rendering fast moving structures such as atrioventricular (AV) and semilunar valves has been limited. Echocardiography has sufficient frame rates to acquire images of moving valves in real-time 3d volumes.

## Methods

We used state-of-the-art commercially-available computer software (Mimics Innovation Suite v17.0, Materialise, Belgium) to develop an efficient work flow that permits importation of 3d echo data and co-registration with MR cardiac angiograms. The AV valve plane is localized using single-shot BFFE pulse sequences prior to MR angiography. After the MR acquisition, an echocardiographic 3d volume of the moving AV valve is acquired (Philips Ie 33 Echo machine, x7-2 matrix 3d probe) and exported to Mimics as DICOM files, 30 phases and 208 slices/phase. Visual inspection of thumbnail images allows phase selection of the fully open valve. Point-to-point registration between the echo-derived AV valve anulus and the MR-derived AV valve plane permits co-registration with the whole heart angiogram. STL files are exported for 3d printing.

## Results

We have successfully combined 3d echo and MR data to create a physical model in order to plan the operative approach for a 2.5 year old child with single ventricle and single AV valve.

## Conclusions

Echocardiographic and cardiac MR imaging technologies each have unique advantages that, until now, have been relegated to separate spheres of diagnostic imaging. Integrating these two modalities to create physical models of complex cardiac malformations will give cardiothoracic surgeons and cardiologists a powerful new tool to plan interventions.

## Funding

Friends of Doernbecher Children's Hospital.

**Figure 1:**
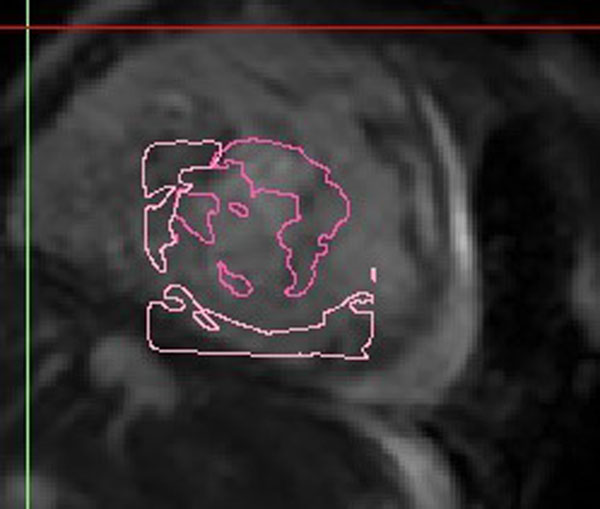
MR AV valve plane and Echo valve outline.

**Figure 2:**
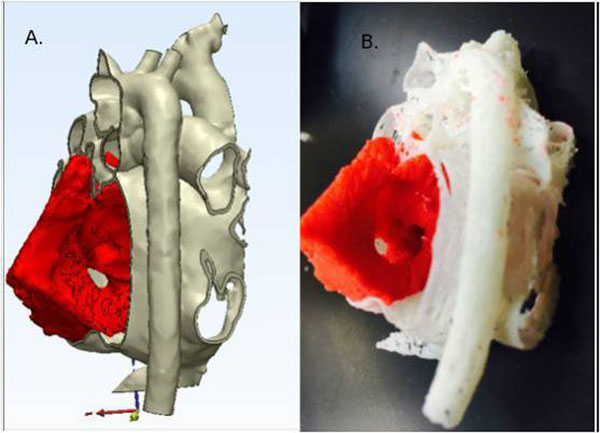
**Integrated 3d Echo (red) and MR angiography (white/gray):** A. Mimics virtual rendering. B. Physical model (MakerBot Industries, Brooklyn NY).

